# Network-based integration of multi-omics data for clinical outcome prediction in neuroblastoma

**DOI:** 10.1038/s41598-022-19019-5

**Published:** 2022-09-14

**Authors:** Conghao Wang, Wu Lue, Rama Kaalia, Parvin Kumar, Jagath C. Rajapakse

**Affiliations:** School of Computer Science and Engineering, Nanyang Technological University, Singapore, 639798 Singapore

**Keywords:** Cancer, Computational biology and bioinformatics

## Abstract

Multi-omics data are increasingly being gathered for investigations of complex diseases such as cancer. However, high dimensionality, small sample size, and heterogeneity of different omics types pose huge challenges to integrated analysis. In this paper, we evaluate two network-based approaches for integration of multi-omics data in an application of clinical outcome prediction of neuroblastoma. We derive Patient Similarity Networks (PSN) as the first step for individual omics data by computing distances among patients from omics features. The fusion of different omics can be investigated in two ways: the network-level fusion is achieved using Similarity Network Fusion algorithm for fusing the PSNs derived for individual omics types; and the feature-level fusion is achieved by fusing the network features obtained from individual PSNs. We demonstrate our methods on two high-risk neuroblastoma datasets from SEQC project and TARGET project. We propose Deep Neural Network and Machine Learning methods with Recursive Feature Elimination as the predictor of survival status of neuroblastoma patients. Our results indicate that network-level fusion outperformed feature-level fusion for integration of different omics data whereas feature-level fusion is more suitable incorporating different feature types derived from same omics type. We conclude that the network-based methods are capable of handling heterogeneity and high dimensionality well in the integration of multi-omics.

## Introduction

Omics refers to the measurements of different molecular entities (e.g., transcriptomics, proteomics, epigenomics, etc.), corresponding to various molecular mechanisms (e.g., genetic, epigenetics, etc.) of a single organism or tissue sample^1^. High throughput ‘omics’ technologies are increasingly being used to decipher underlying molecular mechanisms and invent novel therapeutic strategies for complex diseases such as cancer^2^. Cancer is a microevolutionary process which is contingent upon the localised tissue environment within subjects^3^. The benefits of a personalised medicine approach to the treatment of cancer are increasingly apparent and in turn been adopted by more healthcare practitioners^4^. Initiatives such as ‘The Cancer Genome Atlas (TCGA)’ have curated expansive amounts of multi-omics data from thousands of human subjects^5^. The technological race for high throughput biology has led to a quantum increase in multi-omics data of high dimensions, which requires novel strategies for data analysis. High dimensionality and heterogeneity pose incredible challenges for integration and analysis of multi-omics.

Techniques for multi-omics data analysis can be broadly classified into supervised and unsupervised techniques. Recently proposed supervised techniques include multivariate techniques^6^, group-regularized ridge regression^7^, network-smoothed t-statistic Support Vector Machines (SVM)^8^, generalized elastic net^9^, and deep neural networks^10,11^. However, such approaches require initial filtering and feature selection to reduce data dimensionality or use simple feature integration techniques such as concatenation. They fail to consider interactions among multiple molecular layers measured by different omics technologies. DIABLO, a multi-omics method extending generalized canonical correlation analysis that explicitly takes correlations among different datasets, has been proposed in the supervised framework^12^.

Unsupervised techniques for multi-omics data integration can be broadly classified into joint dimensionality reduction (JDR) techniques and network-based approaches. JDR techniques include sparse Multi-block Partial Least Squares^13^, joint Non-negative Matrix Factorization^14^, Joint and Individual Variation Explained (JIVE)^15^, Multi-omics Factor Analysis (MOFA)^16^, Regularized Generalized Canonical Correlation Analysis (RGCCA)^17^, and tensorial Independent Component Analysis (tICA)^18^. These techniques convert multiple omics datasets jointly into a latent space of lower dimension, capturing biological and technical sources of common variability and disentangling heterogeneities across different omics types. Latent features or factors learned by JDR techniques can be used for a variety of downstream applications such as identification of disease subtypes and patient subgroups, or prediction of clinical outcomes and end points.

**Figure 1:**
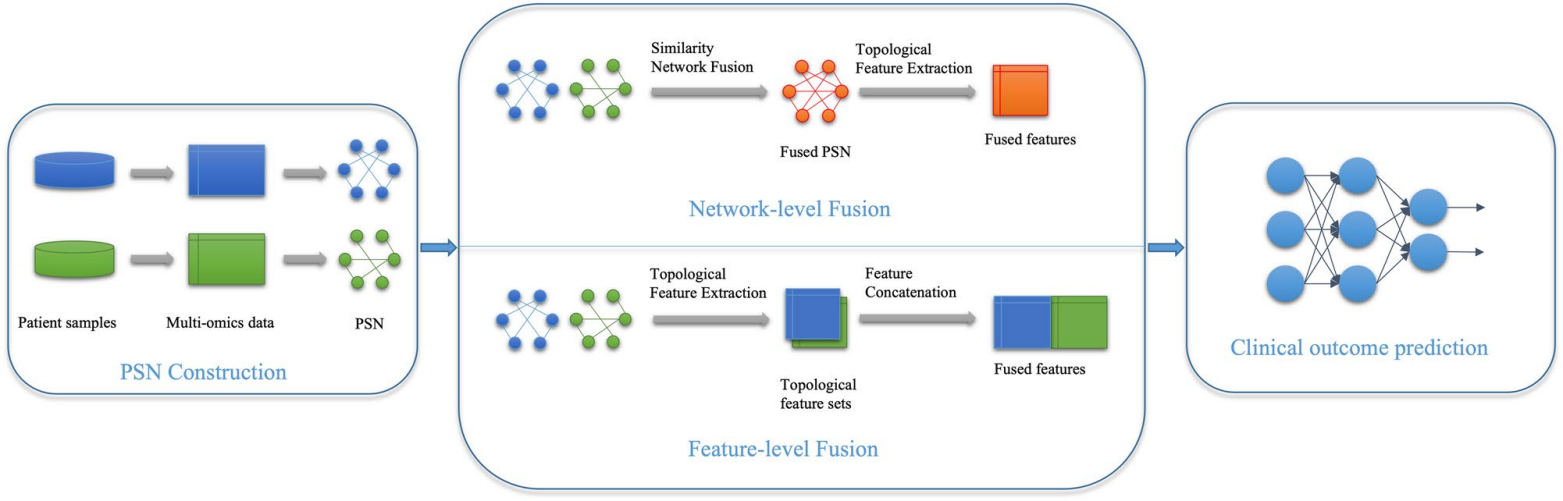
Illustration of our methods for fusion of multi-omics data. Feature-level fusion combines features of patient similarity networks (PSN) and network-level fusion combines PSN derived from individual omics data.

Network-based methods infer relationships between samples/patients or omics features and rely on the networks built using those relations. Examples of networks derived from omics data include Patient Similarity Networks (PSN)^1,19^ or molecular networks such as gene or protein networks. Multiple molecular or patient networks are combined using network integration techniques such as Similarity Network Fusion^20^. Features of integrated networks are then used as inputs to machine learning models for downstream clustering or prediction tasks. The network-based methods rely on network features that are of much lower dimensions than those of omics data and transform heterogeneous omics types into homogeneous networks. The aim of this study is to demonstrate and evaluate two fusion strategies for network-based approaches to multi-omics data integration. Our methods are illustrated in Figure 1. We demonstrate our methods on multi-omics data on an application of clinical outcome prediction in neuroblastoma. This work is an extension of our earlier work for clinical outcome prediction in neuroblastoma by using Support Vector Machines (SVM) and Random Forests^1^, and DNN for single omics data^21^.

Neuroblastoma is a malignancy in developing sympathetic nervous system, which is often accompanied with fatal metastatic disease, resulting in survival rates less than one in two^22^. Treatment of cancer patients depends upon clinical variables like patient’s risk of disease progression or death by disease. Therefore, it is extremely important to predict clinical outcomes of neuroblastoma patients for deciding due course of treatment. By using two multi-omics datasets of neuroblastoma from Therapeutically Applicable Research to Generate Effective Treatment (TARGET) project^22^ and Sequencing Quality Control (SEQC) project^23^, we compare two network-based approaches for integration of multi-omics: network-level fusion and feature-level fusion. For featue-level fusion, multiple network features are extracted from individual PSN and then features from differnt networks are fused; and for network-level fusion, different omics networks are integrated using Similarity Network Fusion (SNF) and then features from the integrated network are used for prediction. Extracted features from PSNs are of much lower dimension to dimensionality of omics features or the sample size.

The features extracted by fusing with omics data are then used as inputs to Deep Neural Network (DNN) classifiers^24^; and Machine Learning classifiers with Recursive Feature Elimination (RFE)^25^. We demonstrate that network-level fusion of multi-omics data outperforms commonly used feature-level fusion on multi-omics datasets and the DNN relevance propagation identifies salient network features better than RFE. In this research, we compare DNN method with four linear classifiers, i.e. SVM (with linear kernel), Random Forests (RF), Logistic Regression (LR), and Decision Trees (DT) as estimators of RFE.

## Materials and methods

### Datasets

We used neuroblastoma multi-omics datasets from the TARGET project^22^ and the SEQC project^23^ to demonstrate applications of our methods. We confirm that all methods were performed in accordance with the relevant guidelines and regulations. Each dataset consists of samples gathered from two omics data types.

- SEQC dataset: SEQC cohort^26^ had a total of 498 neuroblastoma samples, including 176 high-risk and 322 low- and intermediate-risk samples. Microarray and RNA-seq datasets for 498 neuroblastoma patients from SEQC project were downloaded from NCBI GEO database (https://www.ncbi.nlm.nih.gov/gds) with accession numbers GSE49710 and GSE62564, respectively. They both measure gene expression levels but by using different omics technologies.
- TARGET dataset: Target cohort^22^ comprised of 157 high-risk neuroblastoma samples, including gene expression data and DNA methylation data. RNA-seq expression dataset from TARGET project was downloaded from project website (https://ocg.cancer.gov/programs/target/projects/neuroblastoma). And DNA methylation dataset was downloaded from NIH GDC portal (https://portal.gdc.cancer.gov/projects/TARGET-NBL). Gene expression data quantifies the transcriptome in neuroblastoma patients while DNA methylation data (adding methyl groups to genes) signifies epigenomic variations in those patients.

### Patient similarity networks (PSN)

PSN is a graph that represents patients as nodes and similarities between patients as edges and is denoted by $G^m = (V, A^m)$ where *V* denotes the set of subjects and $A^m = \left( a^m_{uv} \right)$ denotes the affinity matrix (the similarity matrix) where $a^m_{uv}$ denotes the similarity of measurements of omics type *m* between subjects $u \in V$ and $v \in V$. If $\phi ^m_v$ denotes omics *m* type measurement of subject *v*, then

1 $$\begin{aligned} a^m_{u,v} = \texttt {sim}(\phi ^m_{u}, \phi ^m_{v}) \end{aligned}$$

where $\texttt {sim}$ is a similarity measure.

The similarity between features of individual omics datasets was determined by the Pearson’s correlation coefficient between the patients:

2 $$\begin{aligned} \texttt {sim}(\phi ^m_u, \phi ^m_v) = \frac{N( \sum _{i} \phi ^m_{u,i} \phi ^m_{v,i}) - \sum _{i} \phi ^m_{u,i} \sum _{i} \phi ^m_{v,i} }{\sqrt{(N \sum _{i} (\phi ^m_{u,i})^2 - (\sum _{i} \phi ^m_{u,i})^2)(N \sum _{i} (\phi ^m_{v,i})^2 - (\sum _{i} \phi ^m_{v,i} )^2)}} \end{aligned}$$

where *N* denotes the total feature number and *i* refers to the $i{\rm th}$ feature in the dataset of omics *m*.

These correlation values were normalized and rescaled to represent positive edge weights by using the Weighted Correlation Network Analysis (WGCNA) algorithm^27^. WGCNA enforces scale-freeness of the PSN by making its nodal degree distribution follow a power law, or at least asymptotically, and thereby our analysis becomes robust to noises and errors.

### Network features

From a PSN, we computed two types of features: centrality features and modularity features. The centrality identifies features giving high scores for most important nodes of the network^28^. We computed 12 centrality features for nodes: weighted degree, closeness centrality, current-flow closeness centrality, current-flow betweenness centrality, eigen vector centrality^29^, Katz centrality^30^, hits centrality^31^ (authority values and hub values), page-rank centrality^32^, load centrality^33^, local clustering coefficient, iterative weighted degree and iterative local clustering coefficient.

Modularity features were extracted by extracting the network modules by clustering the nodal features. We used spectral clustering^34^ and Stochastic Block Model (SBM) clustering^35^ to find network modules and the most optimal number of modules were determined by the silhouette score. Modular memberships of each node to modules were represented by one-hot vectors and the sum of these vectors for all the modules was taken as the modular feature vector for a given node. The centrality features and modular features were concatenated to obtain the network features that were used as the inputs to the classifiers.

### Feature-level fusion

For each PSN obtained from omics dataset *m*, we extracted *n* feature vector $x^m$ for each node or a subject. Using feature-level fusion, we combined individual omics datasets to obtain multi-omics features:

3 $$\begin{aligned} x_v = \underset{m}{\texttt {feature-fusion}} \left( x^m\right) \end{aligned}$$

Feature-level fusion was achieved by concatenating the modularity features and computing the mean of centrality features from individual datasets.

### Network-level fusion

In the network-level fusion, the PSN for multi-omics data *G* is obtained by combining PSN, $G_m$, of individual omics data. We derive the similarity matrix of multi-omics PSN by fusion of those of single-omics PSN:

4 $$\begin{aligned} A = \underset{m}{\texttt {network-fusion}} \left( A^m \right) \end{aligned}$$

We achieved the network-level fusion of single-omics PSN using Similarity Network Fusion (SNF) algorithm^20^.

### Deep neural networks (DNN)

Let us consider $L+1$ layer DNN (feedforward network) for prediction of clinical outcomes where layers $l = 0, 1, \ldots L$ with $l=0$ and $l=L$ denoting the input and output layers of the DNN. Let the output, weights, and biases for layer *l* be denoted as $h^l$, $W^l$, and $b^l$. The input layer receives features *x* from each subject, so $h^0 = x$. For layers $l=1, \ldots L-1$

5 $$\begin{aligned} h^l = f(W^l h^{l-1} + b^l) \end{aligned}$$

*f* denotes the activation function of layer *l*.

The output *y* of the output softmax layer *L* give

6 $$\begin{aligned} y = \texttt {softmax}(W^L h^{L-1} + b^L) \end{aligned}$$

The output class label $k^*$ is assigned the class *k* receiving maximum output activation:

7 $$\begin{aligned} k^* = \underset{k}{\texttt {argmax}} \left( y \right) \end{aligned}$$

The network parameters are learned by minimizing the cross-entropy loss by using gradient descent approach. In our experiments, we used an Adams optimizer to learn the weights and biases of the network.

### Relevance propagation

In order to explore the utility of network features extracted from PSN and the interpretability of our DNN models, Relevance propagation was applied. Relevance propagation is an approach to studying the relevance or attribution of each input feature to a neural network. According to a unified framework comparing existing approaches proposed by M. Ancona et al. (2018)^36^, relevance propagation methods can be classified into perturbation-based and gradient-based methods. Perturbation-based methods compute the relevance of an input feature by simply removing, masking or altering it and comparing the difference with the original output. While the theory of this kind of methods is straightforward, its drawbacks include: (1) slow running time especially with a huge input feature set; (2) unstable results when number of features removed in each iteration varies due to the non-linearity of DNN. On the contrary, gradient-based methods compute the relevance in a single forward and backward propagation through the DNN, which is stable and not time-consuming. Therefore, we adopted a gradient-based method to analyze our DNN model.

Popular gradient-based methods include Gradient * Input^37^, Integrated Gradients^38^, Layer-wise Relevance propagation (LRP)^39^, and DeepLIFT^40^. Notably, Integrated Gradients satisfies two desirable properties, i.e. sensitivity and implementation invariance, while other methods break at least one of them. Sensitivity is satisfied if a feature is given a non-zero attribution when its input and baseline differ and generate different output values. Sensitivity can be readily violated by gradients, when the final prediction is irrelevant to an input and thus always generates a zero gradient regardless of any alterations of the input. Under such circumstances, irrelevant features might be assigned a prominent attribution, which is the condition we attempt to avoid. In addition, implementation invariance means the attributions should be identical to two networks, if their outputs are equal for all inputs, despite that the two networks have disparate implementations. Consequently, we applied Integrated Gradients rather than other approaches because it conforms with sensitivity and implementation invariance.

Specifically, the integrated gradients of the $i{\rm th}$ dimension of an input *x* and a baseline $x'$ can be formulated as^38^

8 $$\begin{aligned} IntegratedGrads_i(x) {:}{:}= (x_i - x_i') \times \int _{\alpha =0}^1 \frac{\partial F(x' + \alpha \times (x - x'))}{\partial x_i} d\alpha \end{aligned}$$

where *F* denotes the function of a DNN, and $\frac{\partial F(x)}{\partial x_i}$ denotes the gradient of *F*(*x*) along the $i{\rm th}$ dimension.

Moreover, several studies have revealed that removing insignificant input features gives rise to performance enhancement^41^. Thus, after computing the attributions of each input features, we recursively removed the features one at a time depending on their attribution ranks, and tracked how the performance changes.

### Recursive feature elimination (RFE)

In addition to DNN, we used Recursive feature elimination (RFE) with other classifier to compare with DNN. RFE is a feature selection method raised by I. Guyon et al. (2002)^25^ designed for identifying salient genes in micro-array gene expression data. RFE utilizes an estimator to rank the features with certain criterion (e.g. linear coefficients), and recursively removes the feature with smallest ranking criterion until a desired feature subset is obtained. The iterative procedure of the RFE algorithm can be depicted as:

1. Train the estimator with the current feature set
2. Rank all the features according to the ranking criterion
3. Remove the feature with the smallest criterion

In this research, we applied four Machine Learning classifiers, i.e. SVM (with linear kernel), Random Forests (RF), Logistic Regression (LR), and Decision Trees (DT) as estimators of RFE. The classifiers were iteratively trained on the network feature set with RFE algorithm to select the paramount features. Our network feature set consists of centrality features and modularity features. We hope that RFE will help discover the centrality features computed by the most suitable algorithms and modularity features representing the vital modules’ membership. Currently, RFE algorithm implemented by Scikit-learn^42^ only supports linear models as estimators.

## Experiments

We analyzed two multi-omics neuroblastoma datasets: (i) microarray and RNA-seq expression datasets from 498 neuroblastoma samples from SEQC project^26^ and (ii) 157 neuroblastoma samples including with RNA-seq expression and DNA methylation datasets from TARGET project^22^. The downloaded datasets were processed by removing any missing values or duplicate values using Pandas and Numpy libraries in Python.

The clinical descriptor used as the label for training DNN classifiers was the binary label ‘death from disease’. By excluding the samples with missing descriptors, we performed binary classification on both data sets: ‘death from disease’ or ‘not’. Both datasets were evaluated using nested 3-fold cross-validation due to relatively limited number of samples.

### Data preprocessing

The Wilcoxon signed-rank test^43,44^ was performed on individual omics datasets to identify the most relevant features of the input features. Correction of multiple test based on Benjamini-Hochberg^45^ was applied to control the false discovery rate, considering the high-dimensional input features. Then the features that were most correlated with the clinical outcome were identified at a significance level (p-value) of 0.001. This effectively reduced the input number of features for each omics datasets. Since gene expression or DNA methylation data include lots of noise and not all the genes/features may be relevant to the disease, Wilcoxon Analysis allowed us to identify and eliminate irrelevant features early, making our models simpler and more accurate.

### Building PSN and feature extraction

Distances between patients were obtained by computing Pearson’s correlation coefficients among omics features and thereby PSNs for each omics dataset were built. The correlation weights were normalized and rescaled to be positive by using WGCNA algorithm^27^, making PSN to behave as scale-free networks. We used the smallest beta value for the algorithm, which achieved 90% of the truncated scale free index. WGCNA algorithm was implemented in house, using Python by applying its formula and rescaling the edges of PSN with the formula while trying different hyperparameters to test if the resultant edges successfully make the PSN scale-free. For network-level fusion, we combined PSNs derived from individual omics datasets via the SNF algorithm^20^, which was implemented using SNFtool library in R.

Network features of PSN were extracted utilizing NetworkX package on Python. Twelve centrality features and modular features were extracted as input features for classifiers. The number of modules detected for each omics dataset were different. In order to discover network modules, spectral clustering was applied using NetworkX and Stochastic Block Model (SBM) was applied using graph-tool package in Python. We extracted 204 modules for microarray and 16 modules for RNA-seq expressions of SEQC dataset, and 60 modules for RNA-seq expressions and 34 modules for DNA methylation of TARGET dataset. For combined networks generated by network-level fusion, 109 and 44 modules were extracted for SEQC and TARGET datasets, respectively. Before being fed into the neural network, the features extracted were normalized to have a zero mean and a unit variance. In order to achieve feature-level fusion, we computed means of centrality features extracted from PSNs of individual omics data, and concatenated the modularity features.

### Training DNN and RFE models

We applied feed-forward DNN for predicting clinical outcomes with features extracted from multi-omics PSNs via Tensorflow V1 framework (https://www.tensorflow.org/versions/r1.15/api_docs/python/tf). The weights and biases of DNN were trained by minimizing the cross-entropy loss function with an Adams optimizer^24^. Notably, the SEQC dataset is extremely imbalanced, where around 77% of the samples belong to the majority class, which is “alive”, while the TARGET dataset does not suffer from imbalance issue. In order to handle data imbalance, we decided to apply weighted cross-entropy loss function^46^ on SEQC dataset, whereas since the data was balanced, we used general softmax cross-entropy loss function on TARGET dataset. The rationale of a weighted cross entropy function is that it assigns different weights to the majority and minority classes to compensate the unbalance naturally. The weightage for class *i* is defined as

9 $$\begin{aligned} weight_i = \frac{\max \limits _{i \in \{0, 1\}}(n_i)}{n_i} \end{aligned}$$

where $n_i$ denotes the number of sample belonging to class *i*.

We used rectified linear unit (ReLU) activation function and dropouts function in the hidden layers. We experimented with batch size of 8 and 32. Early stopping criterion was implemented to determine the convergence of learning in order to avoid overfitting.

Nested cross-validation (CV)^47^ was employed for tuning the hyper-parameters and model selection. Since the number of samples in our dataset is insufficient to create a standalone testing set, using typical cross-validation may lead to overfitting and data leakage, whereas nested CV is designed to address these issues. The algorithm of nested CV is illustrated in Algorithm 1.

The nested cross-validation procedure is composed of outer CV loop and inner CV loop. The training fold for the outer CV is further splitted into k-fold of inner CV. The inner CV loop is similar to the typical CV, which is used for tuning the hyper-parameters such as hidden sizes, learning rates, batch size, etc. The average score of each hyper-parameter set is calculated across all the inner CV folds to discover the best hyper-parameters. Then the outer CV loop is used for model selection, where each model with the best hyper-parameters decided by the inner CV will be tested and compared. This strategy ensures that the testing data for the final evaluation is excluded from the procedures of tuning hyper-parameters, which leads to a more robust evaluation.
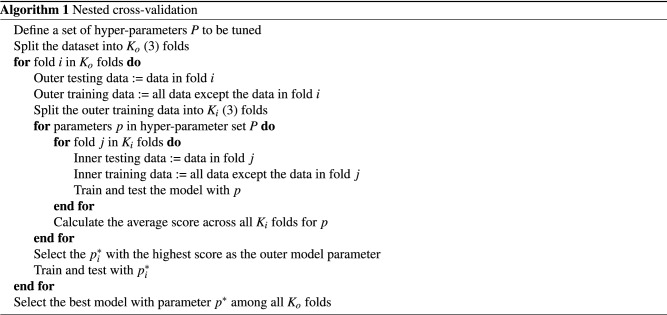


After tuning the parameters and evaluating models, we obtained the DNN model with best performance and then applied Integrated Gradients^38^ on the model to compute saliency scores for input features. Implementation of Integrated Gradients is imported from DeepExplain framework^36^ because DeepExplain supports Tensorflow V1 which we utilized to develop our DNN model. Then the input features were ranked by their saliency scores and removed one by one to seek for performance improvement.

Other than DNN, RFE was also explored for predicting clinical outcomes with PSN network features. We implemented RFE with four machine learning models (i.e. linear kernel SVM, Random Forests, Logistic Regression, and Decision Trees). Network features were evaluated and ranked on the training set, and only the salient features were selected for clinical outcome prediction on the testing set. The details of RFE procedure is explained in Algorithm 2.
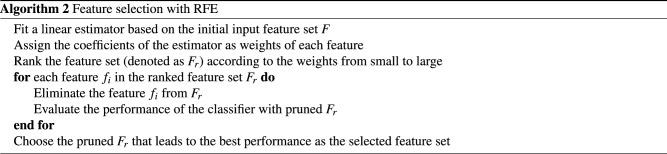


### Comparing with existing methods

To further demonstrate the utility of our approach to integrating multi-omics data and extracting network features, we compared our developed models with several popular approaches, i.e. RGCCA^17^, MOFA^16^, and DIABLO^12^. In our method, the high dimensional and heterogeneous multi-omics data are converted into Patient Similarity Networks (PSN). Then topological features are extracted from the networks, where dimensionality reduction was achieved. And two techniques, i.e. feature-level fusion and network-level fusion, are proposed to integrate PSN or topological features from various omics data. Thus, we compared our method with other approaches that handle feature reduction and multi-omics integration in different ways. RGCCA and MOFA are unsupervised JDR techniques to discovering latent salient factors in omics features that can be readily fed into a downstream analysis. DIABLO is a supervised extension of sparse RGCCA that can be solely applied in a classification task.

### Results

Results of this research consists of DNN performances, Relevance Propagation, Recursive Feature Elimination on Machine Learning classifiers, and comparison with existing multi-omics integration techniques.

#### DNN performances

Survival status predictions with DNN were conducted on SEQC and TARGET datasets. The results are shown in Tables 1 and 2, respectively. Since accuracy fails to measure the performance fairly when the dataset distribution is imbalanced, we also recorded F1 score and ROC-AUC score which works better on ill-distributed datasets. The results are shown in the format of mean ± standard deviations obtained over different random splitting for cross-validation.

**Table 1:** Results of DNN on SEQC dataset.

Dataset	Feature type	ACC (%)	F1 score	ROC AUC	Feature dim
RNA-Seq (GSE49710)	Centrality	74.3 ± 2.4	0.52 ± 0.04	0.79 ± 0.04	13
	Modularity	77.7 ± 8.2	0.56 ± 0.05	0.79 ± 0.05	16
	Both	77.3 ± 5.0	0.55 ± 0.05	0.82 ± 0.03	29
	Abridged	79.8 ± 1.1	0.59 ± 0.03	0.77 ± 0.02	4
Microarray (GSE62564)	Centrality	69.7 ± 12.4	0.19 ± 0.11	0.54 ± 0.04	13
	Modularity	58.9 ± 5.0	0.37 ± 0.08	0.60 ± 0.03	204
	Both	60.1 ± 5.3	0.38 ± 0.06	0.61 ± 0.03	217
	Abridged	62.8 ± 3.9	0.43 ± 0.09	0.66 ± 0.07	133
Network-level fusion	Centrality	68.6 ± 4.8	0.23 ± 0.08	0.54 ± 0.06	13
	Modularity	61.1 ± 4.0	0.37 ± 0.08	0.61 ± 0.02	109
	Both	63.9 ± 4.3	0.36 ± 0.08	0.60 ± 0.03	122
	Abridged	71.3 ± 2.1	0.33 ± 0.06	0.64 ± 0.04	98
Feature-level fusion	Centrality	73.3 ± 2.9	0.49 ± 0.10	0.74 ± 0.03	13
	Modularity	75.1 ± 3.1	0.55 ± 0.06	0.78 ± 0.03	220
	**Both**	**79.7** ± **3.2**	**0.54** ± **0.09**	**0.76** ± **0.04**	**223**
	**Abridged**	**78.9** ± **4.5**	**0.57** ± **0.11**	**0.82** ± **0.04**	**12**

Significant values are in bold.

**Table 2:** Results of DNN on TARGET dataset.

Dataset	Feature type	ACC (%)	F1 score	ROC AUC	Feature dim
RNA-Seq	Centrality	59.4 ± 6.3	0.69 ± 0.05	0.62 ± 0.07	13
	Modularity	61.6 ± 6.8	0.67 ± 0.09	0.62 ± 0.07	64
	Both	60.7 ± 3.5	0.64 ± 0.09	0.64 ± 0.04	77
	Abridged	61.5 ± 4.6	0.66 ± 0.1	0.66 ± 0.05	38
Methylation	Centrality	57.1 ± 6.5	0.36 ± 0.25	0.54 ± 0.09	13
	Modularity	54.1 ± 4.2	0.49 ± 0.19	0.51 ± 0.03	22
	Both	54.6 ± 1.6	0.31 ± 0.11	0.52 ± 0.06	35
	Abridged	54.1 ± 0.1	0.51 ± 0.13	0.53 ± 0.06	34
Network-level fusion	Centrality	57.2 ± 5.2	0.57 ± 0.07	0.62 ± 0.08	13
	Modularity	64.8 ± 8.7	0.65 ± 0.17	0.69 ± 0.07	44
	**Both**	**65.1** ± **4.7**	**0.68** ± **0.04**	**0.71** ± **0.06**	**57**
	**Abridged**	**64.0** ± **5.0**	**0.69** ± **0.05**	**0.77** ± **0.07**	**11**
Feature-level fusion	Centrality	55.7 ± 3.0	0.45 ± 0.29	0.55 ± 0.02	13
	Modularity	60.5 ± 7.9	0.67 ± 0.05	0.61 ± 0.10	55
	Both	61.1 ± 8.0	0.58 ± 0.12	0.61 ± 0.07	68
	Abridged	56.8 ± 8.1	0.64 ± 0.12	0.60 ± 0.15	39

Significant values are in bold.

Firstly, we performed single omics analyses on both datasets to contrast the model performance after multi-omics integration. Then for multi-omics approaches, we recorded results of both feature-level and network-level fusion. For network-level fusion, PSNs of individual omics datasets were fused in accordance with SNF algorithm, and for feature-level fusion, the features of single omics PSNs were averaged or concatenated and fed into DNN. To study the contribution of centrality and modularity features, we also separated the two kinds of network features from the whole feature set, and fed them into DNN models individually. The results are also shown in Tables 1 and 2 when the feature type is centrality or modularity.

Moreover, relevance propagation was applied on optimum models trained on all the datasets to compute the saliency scores of input features. Thereafter, the input features were removed one by one to track the performance variation, and best performances achieved by the abridged feature set are recorded in Tables 1 and 2 together with the feature dimensionality.

In SEQC dataset, over 80% of the samples belong to “alive” class rendering the data distribution extremely imbalanced, while in TARGET dataset, the “alive” and “death” class each takes up around 50% samples. Under such circumstances, we decided to give priority to F1 score while analysing the results regarding SEQC dataset, and focus on accuracy for TARGET dataset, since F1 score balances precision and recall on the positive class and measures imbalanced dataset better.

To optimize the performance of DNN, we applied grid search on hyperparameters to discover the best results under specific configurations. Sizes of hidden layers, number of neurons in the layers, batch size, and learning rate were fixed by experimenting with the validation test. The highest F1 score (0.54±0.09) was achieved on SEQC dataset when the DNN architecture is [8, 64, 4, 8], learning rate is 0.01, and batch size is 8. And on TARGET dataset, best accuracy (65.1±4.7%) was gained with the structure [4, 4, 4], learning rate 0.01, and batch size 32.

As seen from Tables 1 and 2, the experiments of multi-omics dataset with fusion generally achieved higher F1 score or accuracy than prediction based on single omics datasets. On SEQC dataset, highest accurancy (about 80%) and F1 score (around 0.54) were obtained with feature-level fusion technique. Although F1 score of RNA-Sequencing prediction was slightly better, its accuracy is not as good as feature-level fusion. On TARGET dataset, best accuracy (around 65.1%) was achieved by network-level fusion which is better than other techniques. This demonstrates the potential of our approach to integrating multi-omics datasets.

However, it is shown that network-level and feature-level fusion behaved differently on SEQC and TARGET datasets. Notably, the two subsets used for fusion in SEQC dataset, i.e. RNA-Seq and microarray, both belong to the gene expression omics, but leverage different technologies to measure the gene expression profiling. However, in TARGET dataset, RNA-Seq and DNA Methylation data belong to transcriptomics and epigenomics, respectively. It is observed that feature-level fusion is prefered in SEQC dataset, whereas network-level fusion performs better in TARGET dataset. The underlying reason could be that in the condition of homogeneous subsets in SEQC dataset, their features might be redundant rendering the constructed PSNs incompatible to be integrated by SNF algorithm. Therefore, combining the extracted topological features from individual PSNs by averaging the centrality features and concatenating the modularity features is more suitable than network-level fusion. Thereafter, we can leverage the machine learning algorithms to select the features by learning proper weights for them. On the contrary, network-level fusion with SNF is outstanding on multi-omics subsets in TARGET dataset. Consequently, we claim that network-level fusion is generally inclined to better integrate multi-omics datasets, while feature-level fusion is more suitable for combining two homogeneous datasets.

Moreover, on both SEQC and TARGET datasets, models with only modularity features outperformed models with centrality features. This illustrates that a sample’s membership of the modules clustered in the PSN contributed more than a sample’s importance in the whole PSN to the clinical outcome prediction. Nevertheless, a better performance is obtained when both centrality and modularity features are involved in most cases.

#### Relevance propagation results

We applied Integrated Gradients implemented by DeepExplain for computing attributions of input features because DeepExplain can be readily conducted on Tensorflow V1 models. An attribution vector were generated for each sample, thus forming an attribution matrix of size $(n_{sample}, n_{feature})$. In order to generalize the saliency score for each input feature, we calculated the magnitude of the attribution vector across all the samples. Then the input features were removed one at a time according to their ranks of saliency.

In Tables 1 and 2, the Abridged Feature type rows show the performance of DNN after eliminating insignificant features. We found that in the process of removing the input features one by one, the DNN performances did not drop until most features were eliminated. Specifically, for the case of Feture-level fusion on SEQC dataset, when only 12 out of 233 features were preserved, the performance almost maintained the same with the original models. As for Network-level fusion on TARGET dataset, the performance maintained until 11 out of 57 features were left. From the perspective of F1 score, eliminating the irrelevant features even enhanced the performances slightly on both datasets.

Then we investigated about these remained features. The indices of the remained features in SEQC dataset are [25, 171, 125, 202, 51, 211, 118, 87, 14, 13, 15, 18], all of which represent memberships of modules clustered by the spectral or SBM algorithms. In TARGET dataset, the indices are [35, 22, 31, 12, 46, 47, 33, 44, 5, 27, 14]. Two of the remained features belong to centrality features, representing load centrality and iterative local clustering coefficient, and the rest 9 features belong to modularity features.

#### RFE performances

In addition to DNN, we also explored some machine learning techniques’ capabilities of classification with the network features. In the previous section, we compared the performance of DNN models with original input feature set and reduced feature set. In this section, similarly, we would like to compare the performances of several classical classifiers with their performances after Recursive Feature Elimination (RFE). RFE is a technique for feature selection in linear classifiers and is explained in Algorithm 2. The extracted feature set after proposed network-level or feature-level fusion is used for fitting the linear classifiers, and the results with or without RFE feature selection are presented in Table 3 and 4.

**Table 3:** Results of linear classifiers on SEQC dataset.

Fusion strategy	Classifiers	With all features			With RFE feature selection		
		ACC (%)	F1 score	ROC AUC	ACC (%)	F1 score	ROC AUC
Network-level fusion	SVM	61.0 ± 3.2	0.32 ± 0.01	0.49 ± 0.04	78.5 ± 0.3	0.00 ± 0.00	0.55 ± 0.01
	Decision tree	65.5 ± 2.8	0.23 ± 0.04	0.54 ± 0.03	67.0 ± 1.5	0.22 ± 0.04	0.52 ± 0.00
	Random forest	76.7 ± 0.3	0.05 ± 0.04	0.50 ± 0.01	77.5 ± 0.6	0.03 ± 0.04	0.50 ± 0.01
	Logistic regression	63.1 ± 4.2	0.33 ± 0.02	0.47 ± 0.04	78.3 ± 0.0	0.00 ± 0.00	0.57 ± 0.02
Feature-level fusion	SVM	74.7 ± 4.4	0.46 ± 0.08	0.48 ± 0.03	69.9 ± 11.5	0.51 ± 0.02	0.76 ± 0.03
	Decision tree	73.9 ± 2.5	0.39 ± 0.07	0.47 ± 0.02	74.7 ± 2.1	0.37 ± 0.07	0.62 ± 0.05
	Random forest	75.6 ± 0.8	0.25 ± 0.04	0.48 ± 0.01	77.9 ± 2.8	0.31 ± 0.08	0.58 ± 0.05
	**Logistic regression**	73.5 ± 1.3	0.51 ± 0.01	0.53 ± 0.02	**75.3** ± **3.4**	**0.52** ± **0.06**	**0.71** ± **0.05**

Significant values are in bold.

**Table 4:** Results of linear classifiers on TARGET dataset.

Fusion strategy	Classifiers	With all features			With RFE feature selection		
		ACC (%)	F1 score	ROC AUC	ACC (%)	F1 score	ROC AUC
Network-level fusion	SVM	68.2 ± 3.1	0.69 ± 0.03	0.53 ± 0.01	60.5 ± 4.9	0.56 ± 0.10	0.60 ± 0.09
	Decision tree	54.2 ± 4.0	0.51 ± 0.08	0.45 ± 0.03	48.4 ± 1.9	0.47 ± 0.05	0.59 ± 0.07
	Random forest	51.7 ± 4.8	0.42 ± 0.03	0.46 ± 0.05	54.1 ± 6.6	0.51 ± 0.06	0.44 ± 0.05
	**Logistic regression**	**70.1** ± **1.6**	**0.71** ± **0.01**	**0.52** ± **0.06**	61.2 ± 6.1	0.54 ± 0.18	0.68 ± 0.02
Feature-level fusion	SVM	58.0 ± 4.1	0.58 ± 0.01	0.53 ± 0.05	57.3 ± 2.7	0.60 ± 0.03	0.60 ± 0.06
	Decision tree	48.3 ± 3.0	0.50 ± 0.08	0.51 ± 0.05	57.3 ± 3.6	0.60 ± 0.06	0.48 ± 0.03
	Random forest	54.1 ± 5.8	0.55 ± 0.04	0.52 ± 0.05	56.7 ± 0.8	0.53 ± 0.05	0.57 ± 0.02
	Logistic regression	56.7 ± 1.7	0.58 ± 0.04	0.50 ± 0.05	56.7 ± 2.0	0.58 ± 0.02	0.54 ± 0.01

Significant values are in bold.

Table 3 shows the results on SEQC dataset. From the perspective of F1 score, it is apparent that feature-level fusion outperformed network-level fusion to a great extent. The highest F1 scores, achieved by Logistic Regression estimator, are approximate to DNN’s results at around 0.71, when 43 out of 234 features are selected. In Table 4 presenting results on TARGET dataset, we found that the accuracy of network-level fusion is superior to feature-level fusion, which is in accordance with the results of DNN models. The best accuracy is also achieved by Logistic Regression classifier, which is around 70.1%.

As shown in Tables 3 and 4, RFE did not enhance the accuracy and F1 score all the time. In most cases, RFE generated comparable results with the baseline models after removing redundant features, while sometimes it even lowered the performances significantly (e.g. the Logistic Regression case of network-level fusion on TARGET dataset). Therefore, we demonstrate that the feature saliency ranks given by the linear classifiers are not so reasonable as the ones given by relevance propagation of DNN models.

Moreover, it is shown that the performance of Linear classifiers sometimes surpassed DNN’s performance. Best accuracy of Logistic Regression on the TARGET dataset is about 70.1%, while DNN’s accuracy reaches 65.1% at the utmost. However, on SEQC dataset, DNN still outperformed Linear models with RFE slightly.

#### Existing methods

Table 5 displays the comparison of performances obtained by our best model and other popular approaches on SEQC dataset. The three approaches, i.e. RGCCA, MOFA, DIABLO, all aim at reducing the feature dimensionality, discovering the latent factors, and also integrating multi-omics data. Notably, RGCCA and MOFA are unsupervised methods, so we implemented a simple Logistic Regression model for the downstream classification analysis. DIABLO is a supervised method that provides their own function for evaluating performances. As shown, RGCCA is inclined to predict that all the samples belong to an arbitrary label, which leads to unstable prediction with high standard deviation and a low F1 score. A potential reason of its poor performance could be that the selected components are tough to decide when the input feature dimensionality is enormous. MOFA achieves a higher F1 score than our method, but its accuracy is over ten percent lower than ours. DIABLO also yields an inferior accuracy than ours, and unfortunately, DIABLO does not offer any evaluation using metrics of F1 score, which renders it difficult to evaluate its performance on an imbalanced dataset. Generally, our method outperformed the other currently popular approaches on SEQC dataset.

**Table 5:** Comparison of results on SEQC dataset.

Method	ACC (%)	F1 score
RGCCA	58.6 ± 5.9	0.43 ± 0.34
MOFA	67.7 ± 4.5	0.79 ± 0.03
DIABLO	71.1 ± 8.5	–
Ours	78.9 ± 4.5	0.57 ± 0.11

**Table 6:** Comparison of results on TARGET dataset.

Method	ACC (%)	F1 score
RGCCA	43.7 ± 1.5	0.43 ± 0.09
MOFA	52.3 ± 6.0	0.51 ± 0.09
DIABLO	64.2 ± 5.4	–
Ours	70.1 ± 1.6	0.71 ± 0.01

Table 6 shows the comparison of results on TARGET dataset. The best performance of our method given by Logistic Regression model significantly outperformed the other three existing approaches, from both accuracy’s and F1 score’s perspectives. Therefore, we demonstrate that our method is effective in distilling the paramount features for clinical outcome prediction in neuroblastoma.

## Discussion and conclusion

We addressed two challenges, heterogeneity and high dimensionality of multi-omics data, for their integration and analyses by using network-based methods. The multi-omics data were used for building PSNs where nodes represented patients and nodal features of PSNs were used to represent patients’ features. This enables a huge reduction in feature dimensionality - from tens of thousands to tens. Multi-omics data are heterogeneous but the PSNs built with different omics data are homogeneous and can be readily combined since they have similar configurations. Building PSN from multi-omics data allows for both dimensionality reduction and conversion from different omics types to homogeneous networks. Currently, one limitation of this research might be there are only two available omics types in our datasets. In the future, we plan to experiment our approach on datasets with more types of omics data and evaluate the performance of their integration.

We achieved about 79% and 70% accuracies on SEQC and TARGET datasets, respectively, for clinical end point prediction for neuroblastoma, which were significant improvements over the accuracies obtained only with one omics data type. These have important implications practically as the survival rates of neuroblastoma is about 50%. In our experiments, we used only two omics types in the datasets and our methods are generalizable for any number of omics datasets. Our experiments showed that network-level fusion where integration of multi-omics datasets is achieved by fusing homogeneous networks performed better than simply combining features of different PSNs. We used SNF for combining PSNs but one may use other techniques such as tensor based integration. However, when the two subsets both belong to the same omics, e.g. RNA-Seq and microarray in the SEQC dataset, feature-level fusion is prone to generate better results, where the centrality features are averaged and modularity features are concatenated.

The aim for designing DNN relevance propagation and RFE experiments is to identify the paramount network features in the input set. For centrality features, we discovered the most suitable algorithm used for representing a node’s importance in the network. And for modularity features, we identified the essential modules that plays key roles in the clinical outcome prediction. Comparing the baseline feature set with the abridged feature set on DNN, even though the performance is comparative, we realized the majority of the extracted features are insignificant to clinical outcome prediction in neuroblastoma, while only a fraction of the network features is highly related to the task. In the future, we shall investigate more about how these salient features can be extracted and identified in one shot, and how they serve to predict clinical endpoints.

However, scrutinizing the RFE results, we found that RFE strategy failed to enhance the performance, and the selected features varied dramatically in different cases of random splitting, disallowing us to further explore the selected features in a generalized way. Consequently, we believe that relevance propagation in DNN models is more rational than RFE to delve into the networks features extracted from PSN.

As collection of multi-omics data becomes more affordable, novel approaches to omics data integration and analysis are of necessity. By comparing our approach with several existing methods, we demonstrate the potentials of network-based approaches with an application to neuroblastoma clinical outcome prediction. One can further explore our methods on other cancers or complex diseases.

## Data Availability

The datasets used and analysed during the current study are from SEQC and TARGET projects. SEQC project data is available in NCBI GEO database (https://www.ncbi.nlm.nih.gov/gds) with accession number GSE49710 and GSE62564, and TARGET project data is available in NIH GDC data portal (https://portal.gdc.cancer.gov/projects/TARGET-NBL). The implementation of proposed approaches is now released to our GitHub repository (https://github.com/nomoresomethingwentwrong/PSN_Fusion).
